# Patient experience of social and medical fertility preservation fully reimbursed in France

**DOI:** 10.1007/s10815-024-03222-6

**Published:** 2024-08-14

**Authors:** Estelle Hagege, Paul Pirtea, Julie Burette, Anne-Sophie Canepa, Olivier Graesslin, Dominique de Ziegler

**Affiliations:** 1 Department of Obstetrics, Gynecology and Reproductive Medicine, Reims University Hospital Center, Reims, France; 2 Department of Obstetrics, Gynecology and Reproductive Medicine, Foch Hospital, Suresnes, France

**Keywords:** Fertility preservation, Experience, Cost reproductive treatment, Oocyte cryopreservation/ freezing, Social/non-medical/medical egg freezing

## Abstract

**Purpose:**

The purpose of this study is to review patient experience with social fertility preservation (sFP), as compared to medical fertility preservation (mFP), in a context where sFP is fully reimbursed.

**Methods:**

We conducted a retrospective cohort study involving patients who underwent oocyte cryopreservation for mFP between 2017 and 2023 and sFP between 2022 and 2023 at a large ART single center. Additionally, we surveyed patients undergoing sFP and mFP, regarding their experiences, intentions, awareness, and financial consideration.

**Results:**

A total of 97 oocyte retrievals were performed for sFP in 75 women, and 155 were performed in mFP (127 women). Median ages were 36.4 years for sFP and 28.9 years for mFP. Median oocytes retrieved per session were 10 for sFP and 8 for mFP. Ninety-seven percent of of mFP participants were informed by healthcare professionals, while half of sFP participants learned through personal acquaintances. The primary motivation for sFP was a desire for pregnancy while being single. Most respondents in both groups knew that 15–20 oocytes are typically needed for a successful birth. None were aware of the “DuoStim” option, but interest was expressed by most women. Surprisingly, despite full reimbursement for sFP in France, 78% expressed willingness to pay if necessary.

**Conclusion:**

Many women choose sFP due to concerns about declining fertility, often informed by non-medical sources. Free access to sFP can help mitigate the global decline in natality by allowing women to anticipate age-related fertility decline. This study should be considered by other countries as they may increasingly cover sFP costs in the future.

**Supplementary Information:**

The online version contains supplementary material available at 10.1007/s10815-024-03222-6.

## Introduction

The recent availability and high efficacy oocyte cryopreservation by vitrification has enabled fertility preservation (FP) for women, just as it has long existed for men [1]. Practically, FP is envisioned in three distinct circumstances, as reviewed elsewhere [2]. These include women (i) affected by cancer and/or scheduled to undergoing chemo- and/or radiation therapy, (ii) suffering of benign diseases susceptible of affecting ovarian reserve (OR) such as notably, endometriosis and certain auto-immune diseases and, (iii) finally, social FP (sFP), sometimes called elective fertility preservation. The latter, the topic of the present report, primarily encompasses women who are not ready to conceive and fear that further waiting might compromise their chances of childbearing.

The optimal moment for undertaking FP depends on the grounds for which the procedure is performed. In case of mFP, the timing is dictated by treatment imperatives, i.e., chemotherapy/radiation therapy for cancer and surgery for endometriosis. In sFP, the issue is more complex as it is influenced by two opposing factors. First, if sFP is initiated too early, it increases the risk that the cryopreserved oocytes will never be used (because of natural conception occurring in the meantime). Second, if sFP is initiated too late, the response to ovarian stimulation (OS) may be compromised and in turn, the efficacy of the procedure.

The financial cost of sFP is another issue, which further complexifies the choice made by women. In the USA, the common cost for two oocyte retrievals (commonly needed to retrieve 15–20 oocytes) is in the order of $15 K [3]. Certain tech companies, law and consulting firms notably, have generous coverage, but this is the exception rather than the rule. The financial burden of sFP has logically raised issues of cost-effectiveness [4]. Regarding costs, a unique situation now exists in France where sFP has recently become both authorized and fully covered by the insurance system in August 2021. The law, however, restricts access sFP to women aged from 29 to 37 years but does not limit the number of oocyte retrievals that can be performed.

Despite the increasing number of women opting for sFP [5], there is limited data about women’s perceptions of the process, and none currently available in France. The primary motivation for women undergoing sFP is often singlehood or the absence of a suitable partner [6, 7]. An interview-based study highlighted concerns about “panic partnering” [8]. Other studies have indicated that the motivations are not economic or financial reasons, and that most women have a relatively high socio-economic status [9]. A Lebanese study of 402 women indicated that 48.5% had financial concerns regarding the cost of egg freezing [10]. A Belgian study found that women expressed sentiments of injustice due to the lack of reimbursement for sFP [11]. This raises the question of whether the current reimbursement policy in France will change the demographic characteristics of sFP. Furthermore, oocyte vitrification alleviates the pressure associated with the decline in fertility [4, 13]. Some women have reported regrets about prioritizing other projects over their reproductive plans [14]. Freezing oocytes, rather than embryos, offers the advantage of being the person’s sole property [5].

Given that financial factors often hinder access to sFP, studying the outcomes and motivations in the unique context now existing in France offers a genuine interest. With the current wide access to information [6], understanding patients, is of prime interest.

Our study reports the number of oocytes collected for sFP (2022–2023) and mFP data (2017–2023) are provided for comparison. We also conducted a retrospective survey on women’s experience, expectation, and knowledge of issues pertinent to either mFP or sFP.

## Materials and methods

### Study design

This study involved (i) the retrospective analysis of mFP and sFP experience and (ii) replies to online questionnaires sent to patients who underwent sFP (January 2022 to December 2023) and mFP (January 2017 to December 2023).

### Participant selection

The inclusion criterion was one or several oocyte retrievals for FP during the study period. The sFP cohort comprised women who recently (2022) benefited from full reimbursement in France. The mFP cohort included women requiring preservation before treatments like chemotherapy, radiotherapy, or surgery, as well as for endometriosis and reduced ovarian reserve, even if these are less well-defined indications.

### Questionnaire development

Distinct questionnaires (Supplementary Table 1) were designed for the mFP and sFP cohorts. In the sFP group, the questionnaire assessed desires for further oocyte retrievals, future use intentions of cryopreserved oocytes, and whether they would have pursued the procedure without reimbursement. We also enquired about the dual stimulation (“DuoStim”) option, discussed in publications on FP [15–18]. The questionnaire consisted of 16 questions. The questionnaire for the mFP group assessed patient satisfaction with the process, support received, willingness to repeat the procedure, perceived utility, and intentions regarding future pregnancy. This questionnaire included 15 questions (Supplementary Table 1b).

### Data collection

This study reviewed electronic charts of all FP cycles meeting the inclusion criteria at our academic ART center in Reims University Hospital, France, from January 2017 to December 2023. We collected demographic data with patient’s consent of non-opposition. Questionnaires were emailed to women in the sFP and mFP cohorts, with voluntary and anonymized responses ensuring confidentiality and ethical compliance. Data collection spanned several weeks to achieve a sufficient sample size for robust analysis.

### ART procedures

The ART treatment adhered to the protocols of Reims University Hospital. Ovarian stimulation (OS) utilized highly purified urinary menotropins (hMG) and recombinant FSH (rFSH). Individualized doses of hMG (Menopur®, Ferring Pharmaceuticals, Saint-Prex, Switzerland) and/or rFSH (Gonal-f®, Merck, Darmstadt, Germany), ranging between 150 and 450 IU/day, were administered for OS, using a gonadotropin-releasing hormone (GnRH) antagonist protocol. Ovarian follicle development was monitored via transvaginal or suprapubic ultrasonography. Hormonal adjustments were made if necessary to achieve an optimal ovarian response. A GnRH antagonist (Cetrorelix® 0.25 mg, Merck France) was introduced between the fifth and seventh day of stimulation, based on individual responses to OS.

Final oocyte maturation was induced using a combination of human chorionic gonadotropin (hCG) (Ovitrelle® 250 μg, Merck France) and GnRH agonist, triptoreline Decaptyl® (2 × 0.1 mg, Ipsen France), or triptoreline alone (3 × 0.1 mg) if there was a risk of ovarian hyperstimulation syndrome, when ≥ 3 mature follicles of ≥ 17 mm or more in diameters were present on the vaginal ultrasound. Transvaginal or transabdominal oocyte retrieval was conducted 36 h after ovulation trigger.

### Statistical analysis

Quantitative data obtained from the questionnaires were subjected to statistical analysis using appropriate measures of central tendency, dispersion, and comparative analyses. Descriptive statistics were utilized to summarize demographic characteristics and to compare responses between the sFP and mFP cohorts. Continuous variables are presented as median (interquartile range: 25th percentile, 75th percentile) while categorical variables are presented as number of women (percent). The statistical test used for group comparisons was the Student’s *t*-test, as the quantitative variables had a continuous distribution. A *p*-value < 0.05 was considered significant. All analyses were performed using Python (scipy.stats).

### Ethical considerations

This study received approval from the Institutional Review Board at Reims University Hospital, ensuring adherence to ethical guidelines and patient confidentiality throughout the research process (reference number MR00419012024).

## Results

In our ART unit, 75 women aged 29 to 37 years underwent sFP, totaling 97 oocyte retrievals. Additionally, 127 women aged 12 to 40 years underwent mFP, with 157 oocyte retrievals. The median age was significantly higher in the sFP group (36.4 years) compared to the mFP group (28.9 years) (Table 1). The median number of harvested MII oocytes per retrieval was comparable between the groups. The proportion of patients with more than 15 oocytes on their first oocyte retrieval, a recommended figure for FP, was low, at 17.3% and 13.3% in the sFP and mFP groups, respectively. In the sFP group, 20 patients underwent two oocyte retrievals (median 16 MII oocytes), and two patients had three retrievals (median 26.5 MII oocytes). In the mFP group, 24 patients underwent two retrievals (median 10.5 MII oocytes), and four patients had three retrievals (median 17.5 MII oocytes).

**Table 1:** Baseline characteristics of the participants

	sFP (*n* = 75)		mFP (*n* = 127)		
	Median or *N*	% or interquartile	Median or *N*	% or interquartile	*p* value
Age (years)	36.4	[34.0, 37.0]	28.9	[24.0, 34.0]	< 0.001
BMI (kg/m^2^)	21.72	[20, 24.1]	22.5	[20.8, 25.7]	0.022
Tobacco					
Yes	16	21.3%	23	19%	
No	54	72%	94	77.6%	
Past smoker	5	0.07%	4	4%	
FSH (IU/L)	6	[5.2, 7.1]	7	[3.4, 7.7]	0.93
AMH (ng/mL)	2.44	[1.3, 3,1]	4	[2, 6]	0.003
Baseline antral follicle count (*n*)	15	[12, 22]	10	[8, 14]	< 0.001
Total dose of stimulation (IU)	3000	[2437.5, 3562.5]	2925	[2250, 3600]	0.19
E2 levels (pg/mL)	1668.5	[1223.5, 2491.5]	1306	[915.3, 2135.5]	0.082
Number of oocytes retrieved/retrieval	10	[8, 16]	8	[6, 13]	0.12
Number of mature oocytes/retrieval	8	[5, 12]	7	[4, 10]	0.31
Oocyte maturation rate	77.7%		81.5%		
Number of patients > 15 oocytes on their 1st oocyte retrieval	N = 13	17.3%	*N* = 17	13.3%	
^a^Number of oocytes retrieved/patient	14	[9, 21.5]	10.5	[5, 15]	< 0.001
Number of mature oocytes/patients	11	[6, 16]	8	[3.8, 12.3]	< 0.001
Cumulative number of mature oocytes from the first and second oocyte retrievals	16	[13, 19.8]	10.5	[6, 16.3]	0.005
Cumulative number of mature oocytes from the first, second, and third oocyte retrievals	26.5	[26.3, 26.8]	17.5	[12.3, 22.3]	0.26

^a^In the sFP, 20 patients underwent two retrievals and two underwent three retrievals. In the mFP, 24 patients underwent two retrievals and four underwent three retrievals

In the mFP group, there were cancer-related (*n* = 62, 48.9%) and non-cancer-related cases (*n* = 65, 51.1%) (Supplementary Fig. 1). Among cancer cases, hematological cancers accounted for 44%, followed by breast cancers at 29%, and bone cancers at 13%. For non-cancerous causes, the breakdown included 54% due to endometriosis (8% prior to surgery), 28% attributed to a diminished ovarian reserve, and 9% related to immunological pathologies.

Questionnaires were sent to all patients undergoing sFP and only to 110 out of 127 patients undergoing mFP who were > 18 years of age due to ethical considerations. The response rate was 66.7% (*n* = 50) and 58.2% (*n* = 64) in the sFP and mFP groups, respectively.

- Family situation

Most participants were single or unmarried (74% and 67.2% in the sFP and mFP groups, respectively). All were childless, highlighting the importance of FP in these individuals (Supplementary Table 2).

- Reasons for oocyte preservation—sFP

**Fig. 1:**
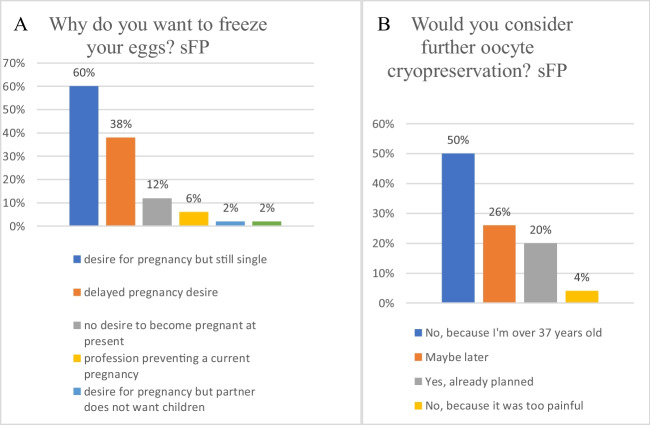
Motivations and preferences in sFP: insights from oocyte cryopreservation surveys

Most respondents (60%) expressed a current desire for pregnancy (Fig. 1A) but indicated their single status as reason for freezing their oocytes. In 38% of cases, women expressed a desire for pregnancy, but favored a long-term rather than immediate plans. Only 6% considered their career as the primary factor justifying their decision to preserve oocytes.

- Willing to pay—sFP

In the sFP, participants demonstrated their acceptance to pay for FP, although this service is currently offered at no cost in France. Indeed, 78% of participants expressed a willingness to pay for FP, emphasizing the perceived value of the procedure.

- Patient intentions for future oocyte retrieval—sFP

In the sFP, half responded that they won’t undergo a second oocyte retrieval (Fig. 1B), attributing their decision to the age limit of 37 years beyond which sFP is not reimbursed anymore, and becomes outlawed, a point addressed in the discussion*.* Among the remaining 50%, 26% indicated a possibility of considering a new retrieval later, and 20% confirmed that such plans were enacted.

- Patient satisfaction with oocyte freezing process

Satisfaction rates were 94% in the sFP group and 90.6% in the mFP group (Supplementary Fig. 2). Additionally, in sFP, when asked if they obtained enough oocytes (Supplementary Fig. 3), the majority (54%) responded negatively. This highlights the mindset of this group, who aims at securing a maximum number of oocytes for their best chances of fertility.

- Source of awareness regarding oocyte freezing purposes

A vast majority, 97%, of mFP participants were informed about oocyte freezing through healthcare professionals (gynecologists, oncologists, or general practitioners) (Table 2). In contrast, half of the participants in sFP learned about oocyte freezing through friends and/or family members. A notable 16% became aware through social media networks and 16% others from television or radio broadcasts.

- Knowledge and preferences in FP

**Table 2:** Sources of information and knowledge on patient awareness of sFP and mFP: insights from survey responses

	sFP (*n* = 50)	mFP (*n* = 64)
^a^How did you learn about the possibility of freezing oocytes?		
Gynecologist	18%	53%
General practioner	4%	3%
Oncologist	0%	33%
Other healthcare	10%	17%
Acquaintance/friend	50%	3%
Media and social networks	32%	0%
Do you know the number of oocytes needed on average to reasonably expect a live birth?		
Yes	64%	59.4%
No	50%	40.6%
According to you, this number is as follows:		
5 oocytes	8%	10.9%
10 oocytes	30%	42.2%
15–25 oocytes	58%	45.3%
> 25 oocytes	4%	1.6%
Would you have liked being able to do double stimulation or “DuoStim” which provides twice as many eggs in just 28 days?		
Yes	70%	50%
No	30%	50%
Have you heard about this possibility of double “back-to-back” stimulation or “DuoStim”?		
Yes	0%	1.6%
No	100%	98.4%
Do you know about the possibility of single parented thought a sperm donation?		
Yes	76%	*Not questioned*
No	24%	*Not questioned*

^a^This question was multiple choice

Both groups had a relatively good understanding of the number of eggs required for securing a future live birth (Table 2). Women undergoing sFP had a higher inclination toward preserving a maximum number of eggs, with 58% knowing that 15–20 oocytes on average are necessary for a live birth. Conversely, a lower fraction of women undergoing mFP (45.3%) were aware of this fact.

A percentage of 1.6% in the mFP and none in the sFP group were aware of the “DuoStim” option. Most of the surveyed women in both groups expressed a vivid interest for this approach (70% and 50% for sFP and mFP, respectively). Interestingly, 76% of women in the sFP group knew that ART was possible for single women (with sperm donation).

- Intentions and perspectives regarding the use of preserved oocytes

**Fig. 2:**
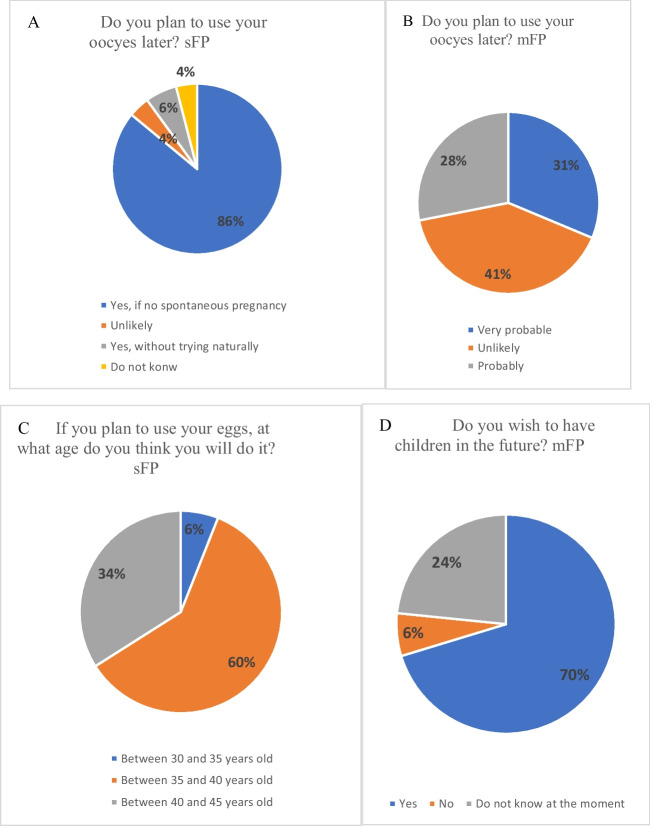
Intentions and perspectives on the use of preserved oocytes

In sFP, 86% responded that they would use oocytes if natural conception were not successful (Fig. 2A). Regarding the age at which they planned to use the frozen oocytes (Fig. 2C), 60% indicated between 35 and 40 years, and 34% between 40 and 45 years.

In the mFP group, the responses were heterogeneous (Fig. 2B). In all, 31% indicated that the future usage of their oocyte was “very probable,” 40.6% “probable,” and 28% “unlikely.” When asked about their desire for children in the future (Fig. 2D), 70.3% responded “yes,” while 23.4% were uncertain at the moment.

## Discussion

To the best of our knowledge, this is the first report of women’s experience with sFP undertaken in a fully reimbursed context. The most striking point revealed by this survey lies in the different expectations voiced by women undergoing sFP and mFP. The quest for sFP is primarily driven by both a lack of partner/immediate conception project and concerns about a possible decline of future fertility. Hence, the desire of these patients is to collect a maximum number of oocytes to ensure an effective backup for their future reproductive projects. Conversely, women undergoing mFP express an ambivalence regarding their desire to have children. This is revealed by heterogeneous responses regarding their intentions to use their cryopreserved oocytes, highlighting divergent attitudes and perspectives toward FP.

Our survey also revealed that no patients in either group had heard of the “DuoStim” option. The latter has been designed for maximizing the number of oocytes cryopreserved over a relatively short period of time with two back-to-back stimulations during the follicular and ensuing luteal phase [19, 20]. Our own experience with “DuoStim” revealed that women perceived it as a longer stimulation rather than two distinct procedures [16]. Moreover, the “DuoStim” option comes handy when there is a time constraint for FP such as notably, in case of breast cancer. To this date, the DuoStim has been proven both efficient and safe [18, 21]. Due to the French law with an age limit of 37 years, the protocol offers useful time-saving but has limitations. Tocci et al. [22] do not support the clinical utility of DuoStim, arguing that the risks and the long-term follow-up of babies born from DuoStim in routine indications are lacking.

In France, sFP is 100% covered until the age of 37 years but outlawed thereafter. Putting an age limit on the possibility of undergoing sFP is not practiced in other countries and opens an ethical debate on this policy. Since 2012, the reimbursement debate has remained active. Mertes et al. [23] argued that in a system where IVF is reimbursed, it is inconsistent to cover IVF treatments with donor eggs for women infertile due to aging, while not allowing the use of their own previously banked eggs for treatment. De Proost et al. advocated for reimbursement, stating that the decision to freeze eggs should not depend on income [24]. Our survey indicated that most patients (78%) would have been willing to pay for sFP. In an American study of 1000 women of reproductive age, women likely to consider egg freezing would be willing to pay $3811 [25]. Allowing women the option of sFP at older ages, possibly without reimbursement, seems a reasonable approach, despite concerns about decreased oocyte quality with advancing age. Women should be made aware of these facts, but if they are willing to cover the costs, they should have the option, similar to the allowance for mFP in older women.

sFP has undergone a profound evolution in a relatively short period of time. In 2013, the ASRM practice guidelines approved the use of egg freezing solely for medical purposes but cautioned against sFP due to limited data on safety, efficacy, emotional risks, and cost-effectiveness [26]. This stance has since shifted. The new Ethics Committee opinion published by ASRM in 2018 now states that sFP is an ethically permissible medical treatment that enhances women’s reproductive autonomy and promotes social equality [27].

Our results highlights the fact that patients seeking sFP often rely on information from their social circles, as is the case in other studies [6]. In a British study of 5482 participants, 73.2% reported an awareness of sFP [28]. This figure is 87.2% in an American study [25] and 65% in a Lebanese study [10], showing that awareness and knowledge of FP are higher than in studies conducted over a decade ago. Most women indicated in our survey that they had been informed about sFP by friends, relatives, social media, and non-specialized websites. Interestingly, only 30% of our study participants had been informed by healthcare professionals about sFP, reflecting a gap in physician–patient communication. Poor perception of the adequacy of counseling has been shown to increase decision regret following sFP [29], highlighting the critical importance of comprehensive explanation and emotional support throughout the preservation process.

Our study findings align with previous research regarding the primary reason for sFP, which points at the primary cause being the lack of partner, or immediate conception project [6, 7]. Some studies among student populations have identified career aspirations as the most prevalent reason for undergoing sFP [30, 31], contrary to our study which found professional reasons in only 6% of cases. However, these studies were undertaken in women in their early twenties, which is not reflective of the population who typically undergo sFP. Given the median age of 36 in our study, and the context where the majority had a higher education degree in other studies [9, 32], it is highly likely that our population has already achieved their career goals sufficiently. Our data also indicate that women are more inclined to wait for the right partner before starting a family rather than embarking in a single parent project with donor sperm. In another study, women preferred not to use sperm donation for FP [12], opting instead for sFP to extend their search for the right partner and optimal conception opportunities. This approach helps alleviate the pressure women feel in their thirties due to fertility decline concerns [4, 13].

Our observation is not unique, as the upward trend for sFP demand is global. ASRM [33], for example, reported in 2021 a 31% increase in requests over the previous year. Other countries observed similar results notably, Germany [34] and Belgium [35].

There is mounting evidence that the reproductive efficacy of previously frozen oocytes is generally equivalent to that of fresh oocytes. Multiple studies have reported that implantation rates of frozen oocytes are comparable to that of fresh oocytes [36–41]. One study including 422 autologous cryopreserved oocyte cycles, 93,181 autologous fresh oocyte cycles, 2223 donor cryopreserved oocyte cycles, and 9691 donor fresh oocyte cycles reported no difference in cancellation, implantation, pregnancy, miscarriage, or live birth rates between autologous fresh and cryopreserved oocytes [42].

For sFP, Cobo et al. reported that on average 15 frozen oocytes are necessary to achieve a live birth when freezing takes place before the age of 35 years [43, 44]. Goldman et al. [44] predict, however, that 33 frozen oocytes would be necessary at 40 years of age. These numbers are important to take into account, as several reports indicated that the median age at the time of sFP is of approximately 38 years in Australia [12], the UK [45], and the USA [46]. As eloquently said by Dr. Marcelle Cedars, sFP is not “a baby in the freezer, but a chance to possibly get pregnant” [47]. Indeed, patients may harbor overly optimistic expectations regarding planned oocyte cryopreservation, as evidenced by a survey indicating that numerous fertility patients of advanced reproductive age (43 to 45 years) overestimate their likelihood of conception [48].

The issue of possible diminished ovarian reserve is a complex one when considering sFP. While diminished ovarian reserve does not amount to decreased natural fertility [49], accumulating ≥ 15 vitrified oocytes may be challenging. Further work is needed to clarify this issue.

sFP may play a role for mitigating the decline in natality, which particularly affects all European countries and certain Asian countries such as notably, China and Japan. In all these countries, fertility rates are all below population replacement level. As delaying the reproductive project is a primary factor in the decreased fertility observed in these countries, sFP could be instrumental in minimizing this effect. Responses to our survey certainly speak in favor of this hypothesis. In that sense, the full reimbursement of sFP may be a judicious measure for countries that wish to palliate the global decrease in fertility. France, by pioneering this measure, may therefore constitute an example to follow. France is indeed a good model when it comes to supporting overall fertility. One common metrics used for assessing the global effect of measures undertaken for supporting fertility is the gap between the number of children desired and actual number of children in the family. A low value—France has the lowest recorded—reflects good efficiency of all the measure taken for favoring fertility [50]. Evidently, it would be crucial to measure the impact of reimbursing sFP on this parameter of fertility support.

Published evidence offers reassurance regarding the safety of ovarian stimulation on future fertility and the risk of cancer [51]. In a retrospective study on 4052 donor oocytes retrievals, Bodry et al. reported a law rate of complications, 17 patients (0.42%) related to oocyte retrieval and moderate/severe ovarian hyperstimulation syndrome in 22 patients (0.54%) [52, 53]. Moreover, the classical major risk of stimulation OS—ovarian hyperstimulation syndrome—has now been practically eradicated by the possibility of triggering ovulation with an agonist of GnRH [54].

The primary strength of our study is that it represents the first comprehensive evaluation of sFP in a country where the procedure is fully reimbursed. Furthermore, we obtained the viewpoints of these patients and compared them to those of women who underwent mFP. The potential role of sFP for mitigating the global decline in fertility makes this experience of paid sFP most crucial. One of the lessons learned is that sFP should be considered at an earlier age than what is now authorized in France in order to optimize its efficacy.

The limitations of our study on sFP are the rather small sample size, with 50 and 64 responses to questionnaires in the sFP and mFP groups, respectively. Additionally, our data were collected from a single ART center. Furthermore, the questionnaire’s nature, which primarily consisted of single or multiple choice questions rather than in-depth interviews, may have limited the patients’ ability to express fully their viewpoints.

## Conclusion

Our study highlights the outstanding differences in perceptions—knowledge, expectation, etc.—between sFP and mFP. Our survey indicates that most patients opt for sFP due to concerns about declining fertility in the absence of an immediate conception project. Conversely, women undergoing mFP express ambivalence regarding their desire to have children, leading to diverse responses concerning their intentions to use their cryopreserved oocytes. The recent reimbursement of the procedure in France has predictably stimulated a marked increase in demand for sFP. Request for sFP surged in France and elsewhere and parallels the global trend of delayed fertility projects. Availability and free of charge sFP are important in light of the current global decrease in natality, which is far below replacement level in most European countries and notably, China. Free access to sFP may slightly reduce the dwindling of fertility expressed as number of children per women. Easy access to sFP is therefore a measure, which we see as beneficial for countries facing decline in natality and population dwindling. The optimal number of oocytes retrieved, and retrievals needed must be individualized as a function of patient preferences, age, and ovarian reserve. Furthermore, given that patients are well-informed through rapid access to information in our society (social media, television, peers), proactive counseling on these decisions is crucial.

## Supplementary Information

Below is the link to the electronic supplementary material.

Supplementary file1 (DOCX 84.2 KB)

## Data Availability

Data will be available for request.
