# Supplementary material for: Patient experience of social and medical fertility preservation fully reimbursed in France
Source: J Assist Reprod Genet. 2024 Aug 14;41(10):2813–22. doi: 10.1007/s10815-024-03222-6 (PMC11534921; doi:10.1007/s10815-024-03222-6)
Supplement: Supplementary file 1 — Supplementary file1 (DOCX 84.2 KB) [file 10815_2024_3222_MOESM1_ESM.docx]

| 1. What age were you on the day of your fertility preservation? |
| --- |
|  |
| 1. Are you: |
| - - In a relationship with a man |
| - - In a relationship with a woman |
| - - Single |
| 1. Do you already have children? |
| - - No |
| - - Yes |
| 1. How did you learn about the possibility of freezing oocytes? |
| - - General practitioner |
| - - Gynecologist |
| - - Other healthcare |
| - - Acquaintance / Friend |
| - - Media and Social Networks |
| - - Other: please specify |
| 1. Why do you want to freeze your eggs? |
| - - No desire to become pregnant at present |
| - - Desire for pregnancy but still single |
| - - Desire for pregnancy but partner does not want children |
| - - Profession preventing a current pregnancy |
| - - Delayed pregnancy desire |
| - - Do not know |
| 1. Would you have undergone egg freezing if it cost: |
| - - 4,000 euros |
| - - 3,000 euros |
| - - 2,000 euros |
| - - 1,000 euros |
| - - 500 euros |
| - - None of the above options |
| 1. Are you satisfied with your experience? |
| - - Yes |
| - - No |
| 1. Would you consider further oocyte cryopreservation? |
| - - Yes, it's planned |
| - - Maybe later |
| - - No because it's too painful |
| - - No because past the age of 37 |
| - - No for medical reasons |
| 1. Do you think you obtained enough eggs? |
| - - Yes |
| - - No |
| 1. Would you have liked being able to do double stimulation or “DuoStim” which provides twice as many eggs in just 28 days? |
| - Yes |
| - No |
| 1. Have you heard about this possibility of double ‘back-to-back' stimulationor “DuoStim”? |
| - Yes |
| - No |
| 1. Do you know the number of oocytes needed on average to reasonably expect a live birth? |
| - Yes |
| - No |
| 1. According to you, this number is: |
| - 5 eggs |
| - 10 eggs |
| - 15-25 eggs |
| - >25 eggs |
| 1. Do you plan to use your oocytes later? |
| - Unlikely |
| - Yes if no spontaneous pregnancy |
| - Yes without trying naturally |
| - Do not know |
| 1. Do you know about the possibility of single parented thought a sperm donation? |
| - Between 30 and 35 years old |
| - Between 35 and 40 years old |
| - Between 40 and 45 years old |
| 1. Are you aware of the possibility of planning a pregnancy as a single woman with sperm donation? |
| - Yes |
| - No |

**Supplementary Table 1a: Questionnaire for sFP**

| 1. What was your age on the day of your fertility preservation? |
| --- |
| 1. Are you: |
| - - In a relationship with a man |
| - - In a relationship with a woman |
| - - Single |
| 1. Do you already have children? |
| - - No |
| - - Yes |
| 1. How did you learn about the possibility of freezing oocytes? |
| - - Oncologist |
| - - General practitioner |
| - - Gynecologist |
| - - Other healthcare |
| - - Acquaintance / Friend |
| - - Other: please specify |
| 1. Does your partner/family support you in this choice to freeze your eggs? |
| - - Yes |
| - - No |
| 1. What is the underlying condition justifying this egg freezing? |
| - - Breast cancer |
| - - Another type of cancer |
| - - Decreased ovarian reserve |
| - - Endometriosis requiring surgical intervention for pain |
| - - Endometriosis requiring surgical intervention for other reasons (Cysts?) |
| - - Previously operated endometriosis |
| - - Endometriosis without planned surgical intervention |
| - - Another non-cancerous condition |
| 1. Are you satisfied with your experience? |
| - - Yes |
| - - No |
| 1. How did you feel about the egg freezing process? |
| - - Reassured |
| - - Adequate |
| - - Not enough explanation |
| - - Not enough support |
| - - Psychologically painful |
| - - Physically painful |
| 1. Would you consider further oocyte cryopreservation? |
| - - Yes |
| - - No for medical reasons |
| - - No because I do not want to |
| - - Do not know |
| 1. Would you have liked being able to do double stimulation or “DuoStim” which provides twice as many eggs in just 28 days? |
| - Yes |
| - No |
| 1. Have you heard about this possibility of double ‘back-to-back' stimulationor “DuoStim”? |
| - Yes |
| - No |
| 1. Do you know the number of oocytes needed on average to reasonably expect a live birth? |
| - Yes |
| - No |
| 1. According to you, this number is: |
| - 5 eggs |
| - 10 eggs |
| - 15-25 eggs |
| - >25 eggs |
| 1. Do you plan to use your eggs later? |
| - Very likely |
| - Likely |
| - Unlikely |
| 1. Do you wish to have children in the future? |
| - Yes |
| - No |
| - Do not know at the moment |

**Supplementary Table 1b: Questionnaire for mFP**

**Supplementary Figure 1: A. patient’s medical condition on mFP (cancer (n = 62 , 48.9%) versus non-cancer (n= 65 , 51.1%))**

|  | sFP (n=50) | mFP (n=64) |
| --- | --- | --- |
| Heterosexual | 26% | 31.3% |
| Homosexual | 0% | 1.6% |
| Single | 74% | 67.2% |
| Do you have children? | | |
| Yes | 10% | 17.2% |
| No | 90% | 82.8% |

**Supplementary Table 2: Family situation in the two groups**

**Supplementary Figure 2: Patient Satisfaction with Oocyte Cryopreservation**

**Supplementary Figure 3: Assessment of Oocyte Quantity Satisfaction - sFP**
